# Obesity, Leptin and Breast Cancer: Epidemiological Evidence and Proposed Mechanisms

**DOI:** 10.3390/cancers11010062

**Published:** 2019-01-09

**Authors:** Sebastiano Andò, Luca Gelsomino, Salvatore Panza, Cinzia Giordano, Daniela Bonofiglio, Ines Barone, Stefania Catalano

**Affiliations:** 1 Department of Pharmacy, Health and Nutritional Sciences, University of Calabria, 87036 Arcavacata di Rende (CS), Italy; sebastiano.ando@unical.it (S.A.); lugelso@gmail.com (L.G.); sasapanza@libero.it (S.P.); cinzia.giordano@unical.it (C.G.); daniela.bonofiglio@unical.it (D.B.); 2 Centro Sanitario, University of Calabria, Via P Bucci, 87036 Arcavacata di Rende (CS), Italy

**Keywords:** breast cancer, obesity, adipokines, leptin, adiponectin, inflammation, estrogens, insulin/insulin-like growth factor I

## Abstract

The prevalence of obesity has been steadily increasing over the past few decades in several developed and developing countries, with resultant hazardous health implications. Substantial epidemiological evidence has shown that excessive adiposity strongly influences risk, prognosis, and progression of various malignancies, including breast cancer. Indeed, it is now well recognized that obesity is a complex physiologic state associated with multiple molecular changes capable of modulating the behavior of breast tumor cells as well of the surrounding microenvironment. Particularly, insulin resistance, hyperactivation of insulin-like growth factor pathways, and increased levels of estrogen due to aromatization by the adipose tissue, inflammatory cytokines, and adipokines contribute to breast cancerogenesis. Among adipokines, leptin, whose circulating levels increase proportionally to total adipose tissue mass, has been identified as a key member of the molecular network in obesity. This review summarizes the current knowledge on the epidemiological link existing between obesity and breast cancer and outlines the molecular mechanisms underlying this connection. The multifaceted role of the obesity adipokine leptin in this respect is also discussed.

## 1. Introduction

Overweight and obesity are preventable conditions characterized by an abnormal or an excessive fat tissue accumulation that may significantly impair health status.

The World Health Organization (WHO) and the National Institutes of Health (NIH) refer to overweight as a body mass index (BMI, defined as weight (kg)/height (m^2^)) greater than 25 kg/m^2^, and to obesity as a BMI greater than 30 kg/m^2^ (30.0–34.9, grade I; 35.0–39.9, grade II; and ≥40, grade III). The increase in BMI is typically a result of an imbalance between exceeding energy consumption from dietary intake and energy expenditure from physical and metabolic activity. Unfortunately, fueled by major economic growth, social/technological changes, and a transition in nutrition over the last 30 years, the prevalence of obesity has been on the rise alarmingly [1], reaching epidemic proportions in many parts of the world. At present, according to the latest WHO fact sheets, more than 1.9 billion adults and a further 600 million people can be, respectively, classified as overweight or obese worldwide, and these rates are projected to increase at a faster pace in the coming decades [2]. This scenario has drawn particular attention from researchers due to the fact that obesity is associated with concomitant or increased risk of nearly every chronic condition, including diabetes, coronary artery disease, hypertension, kidney disease, disability, and poor mental health [3,4]. Numerous studies have also looked into the link between obesity and cancer development at various sites, especially the pancreas, oesophagus, colorectum, prostate, and breast. Indeed, there is a growing appreciation that the excessive adiposity is correlated with increased cancer incidence at a population level and may lead to poor cancer clinical outcomes [5,6,7,8]. In this regard, an intricate connection between obesity and both breast cancer incidence and the clinical behavior of the established neoplasia has been extensively described.

Here, we will review the complex and not yet completely understood impact of obesity on breast cancer pathogenesis. First, we will provide an update of the recent epidemiological research focused on the association between obesity and breast cancer. Then, we will address the molecular mechanisms by which obesity-associated changes may affect breast malignancy, and we will outline the role of the obesity cytokine leptin in this type of cancer, discussing both clinical and basic research evidence.

## 2. The Epidemiological Association between Obesity and Breast Cancer

Breast cancer is the most commonly diagnosed neoplastic disease and represents the leading cause of cancer-related mortality among women worldwide [9]. According to GLOBOCAN 2012, it was estimated that breast cancer accounts for 25% of cancer cases and 15% of cancer-related deaths, with approximately 1.7 million cases and 521,900 deaths [10]. In addition to established factors (i.e., older age, female sex, family history, gene sequence mutations, reproductive factors), it has been reported that around 20% of breast cancers may be due to modifiable risk factors, including alcohol drinking, smoking, excess body weight, and physical inactivity [11,12,13,14,15,16]. In particular, several studies have shown the relationship existing between obesity status and breast cancer, highlighting the potential to reduce the disease burden through an increase in individual healthy behaviors. Different clinical trials focused on obesity and breast cancer have been conducted or are ongoing (http://www.clinicaltrials.gov); a list of these studies is reported in Table 1 [10,13,17,18,19,20,21,22,23,24,25,26,27,28,29,30,31,32,33,34,35,36,37,38,39].

### 2.1. Increased BMI and Risk of Breast Cancer Occurrence

The effects of obesity on the risk of developing breast cancer have been evaluated among diverse population groups, reflecting important differences across menopausal individuals and disease subtypes. The Women’s Health Initiative Clinical Trial, following 67,142 postmenopausal women aged 50 to 79 years for a median of 13 years, showed that obese postmenopausal women are at a greater risk of developing breast cancer compared with their normal-weight counterparts, especially for women with obesity grades 2 and 3 (BMI >35.0 kg/m^2^) [40]. Obesity grades 2 and 3 were also associated with more advanced disease, such as larger tumor size, lymph-node positivity, and regional/distant stage after diagnosis [40]. A large increase in the risk for postmenopausal breast cancers was also evidenced in a population-based cohort study of 5.24 million U.K. adults [41] and confirmed in many other cohort and case–control studies [5,42,43,44,45]. This association seems to be restricted to estrogen receptor (ER)- and progesterone receptor (PR)-positive breast cancers [40,44,45,46,47], whereas ER-negative and triple-negative diseases are slightly or inversely correlated with obesity after menopause [40,44,47,48,49]. Indeed, a meta-analysis of 39 studies revealed that the relative risk increased by approximately 40% for ER-positive postmenopausal breast cancer and was attenuated for ER-negative cases [45]. More recently, increased BMI was also positively correlated with a higher risk of less aggressive tumor subtypes, as defined by clinically used biomarkers (ER+, PR+, HER2 (Human epidermal growth factor receptor 2)−, Ki67low, Bcl-2 (B-cell lymphoma 2)+, and p53−), among postmenopausal women who were non-users of estrogen–progestin therapy [50].

Conversely, studies among premenopausal obese women are still uncertain and unreliable (reviewed in [51]). On the basis of the epidemiological findings obtained up to 2017, the World Cancer Research Fund (WCRF) and the American Institute for Cancer Research (AICR) report highlighted the strong evidence that being overweight or obese decreased the risk of premenopausal breast cancer. Compared with women who had a BMI less than 21 kg/m^2^, women with a BMI greater than 31 kg/m^2^ exhibited a reduced relative risk in a pooled analysis of prospective cohort studies [52]. Interestingly, a meta-analysis study which included 7930 premenopausal patients showed an approximately 8% reduced risk per 5 kg/m^2^ increase in BMI [5]. One leading hypothesis for the pathophysiology relating obesity to reduced breast cancer risk in pre-menopause may rely on the higher levels of estrogens synthesized in peripheral tissues, along with ovarian estrogen production, that activate a negative feedback mechanism on the hypothalamic pituitary axis and cause a reduced gonadotrophin release [53]. This leads to amenorrhea and a diminution in ovarian activity with a markedly decreased production of progesterone [54], a promoter of proliferation in the breast [55]. However, this likely does not fully explain the lower premenopausal risk, since subsequent studies have hypothesized that progesterone is neutral or even protective for breast cancer [56]. Moreover, a non-significant relationship of BMI with risk of premenopausal breast cancer [57,58] or a modestly positive correlation between obesity and risk [59,60] was observed in other studies. These conflicting results may be likely due to the differences in ethnicities (e.g., black versus white populations) and/or hormonal receptor status. Excessive adiposity correlated with a lower risk of ER-positive breast cancer and with a higher risk of ER-negative breast cancer before menopause [46,48,61,62]. In African American women participating in the AMBER (African American Breast Cancer Epidemiology and Risk) Consortium, historical obesity (defined as young adult weight) was associated with a decreased risk of ER-positive breast cancer, but not overall or ER-negative breast cancer, whereas recent BMI was not significantly linked with pre-menopausal ER-positive, ER-negative, or triple-negative diseases [47].

In addition, it has been reported a positive association between BMI and the risk of developing inflammatory breast cancer, regardless of both menopausal status and ER expression [63,64,65].

Excess body weight also increased the risk of developing a second primary malignancy in breast cancer survivors, most likely due to the fact that obesity represents a risk factor for developing different primary cancers in the general population. In particular, a meta-analysis of 13 prospective, 5 cohort, and 8 nested case-control studies showed that high BMI levels correlated with an increased relative risk of contralateral breast (RR (Relative Risk) = 1.37, 95% CI (Confidence Intervals): 1.20–1.57), endometrial (RR = 1.96, 95% CI: 1.43–2.70), and colorectal (RR = 1.89, 95% CI: 1.28–2.79) cancers in women with a previously diagnosed breast cancer [66].

### 2.2. Increased BMI and Risk of Breast Cancer Mortality

Several studies have reported a correlation of obesity at and after breast cancer diagnosis with mortality risk. A meta-analysis conducted on 43 studies, that enrolled breast cancer patients in the years 1963–2005 (sample size: 100–424,168, median 1192), evidenced a 33% increased rate of death among obese subjects [67]. More recently, Chan et al., through a robust meta-analysis of 82 studies including 213,075 breast cancer survivors with 41,477 deaths, estimated a 41% and a 35% increased risk, respectively, of all-cause mortality and breast cancer-specific mortality for obese women compared to their normal-weight counterparts [68]. This association was greater 1 year or more after diagnosis compared to less than 1 year after diagnosis, highlighting a persistent link between obesity and poor outcomes over time. In addition, for each 5 kg/m^2^ increase in BMI, it was demonstrated an approximately 18% increment in the risk of total and breast cancer mortality. Conversely, it was shown that only the hazard of overall mortality, but not of breast cancer-specific mortality, differs for weight gainers in respect to weight maintainers [69]. Interestingly, in contrast to the stronger breast cancer risk in post-menopausal and ER-positive obese patients, the relationship existing between BMI and mortality was evident in both premenopausal (RR = 1.75, 95% CI 1.26–2.41) and postmenopausal (RR = 1.34, 95% CI 1.18–1.53) settings [68]. Other meta-analyses have also reported that the obesity effect was numerically larger in premenopausal than in postmenopausal women, although no statistically significant difference was found in these two groups (HR (Hazard Ratio) of 1.47 versus 1.22, [67] and 1.23 versus 1.15, [70]). There was no evidence on the differential interaction of obesity and mortality in relation to hormone receptor status of cancer [68,70].

### 2.3. Waist-To-Hip Ratio (WHR) and Breast Cancer

BMI has important limitations due to its the incapability to differentiate between fat and lean body mass as well as to measure fat distribution in the body. Therefore, alternative tools to define obesity status, including waist circumference (WC) and WHR, have been proposed in some studies. In the meta-analysis of Protani, poorer overall (HR = 1.33) and breast cancer-specific (HR = 1.33) survivals were shown in obese compared with non-obese subjects, stratified by WHR [67]. Elevated WC and WHR have been also associated with increased risk for ER-positive/PR-positive postmenopausal [44,48,71] and triple-negative premenopausal breast cancers [48].

### 2.4. Obesity and Therapeutic Implications

In addition to influencing breast cancer occurrence and mortality, obesity status has profound implications on therapeutic management of patients. Certainly, medical imaging and image-guided intervention may represent a challenge in the obese population; on the other hand, difficulties to likely accomplish the standard oncologic care using surgery, radiotherapy, chemotherapy, and/or endocrine therapy may be also encountered.

It has been reported that morbid obesity was significantly associated with an increased risk of perioperative and medical complications after mastectomy with or without breast reconstruction surgery. Patient complications may include wound dehiscence, prosthetic/flap failure, infections, sepsis, deep venous thromboembolism, pneumonia, lymphedema, and return to operating room [72,73,74,75,76].

Large breast size, which is commonly observed in women with increased BMI, also represents an important factor determining the occurrence of late adverse effects and poor cosmetic outcomes in patients after breast conserving surgery and adjuvant radiation treatment [77,78,79]. Interestingly, relationships between overweight/obesity and radiotoxicity risk seem to be more evident in women with endothelial nitric oxide synthase (eNOS) and myeloperoxidase (MPO) genotypes associated with higher levels of nitric oxide and reactive oxygen species (ROS) [80]. However, a study on a cohort of 110 patients with a median BMI of 33.6 kg/m^2^ revealed that using prone position for three-dimensional conformal radiotherapy (3D-CRT) to deliver post-lumpectomy whole breast irradiation resulted in favorable toxicity profiles and excellent cosmesis rates [81].

Dosing of chemotherapy among obese women with breast cancer is actually an important issue related to treatment decision-making. Indeed, there are several data showing that the worst clinical outcomes observed in obese patients may be justified by under-dosing of cytotoxic therapies worldwide [82,83]. For instance, a retrospective analysis of four randomized trials conducted by the International Breast Cancer Study Group, assessing adjuvant chemotherapy (cyclophosphamide, methotrexate, and 5-fluorouracil) in premenopausal women with node-positive breast cancer, revealed that obese patients who received a lower chemotherapy dosage (<85% of the expected administration) experienced a significantly higher relapse frequency and a reduced survival rate [84]. Unfortunately, it has been estimated that up to 40% of obese subjects undergoing chemotherapy for breast cancer received considerably reduced doses as compared to those that should be administered if actual body weight was considered in dose calculations [82,84]. Therefore, the American Society of Clinical Oncology (ASCO) Practice Guidelines is recommending to avoid unnecessary dose reductions in obese patients, especially when treatment is given with curative intent [85]. The rationale for these guidelines is based on observational and retrospective studies confirming the safety and the importance of delivering full weight-dosed chemotherapy [85,86]. As early as 1996, Rosner and colleagues reported that obese patients, who received weight-based doses of the most dose-intensive cyclophosphamide, doxorubicin, and fluorouracil (CAF) regimen according to their actual weight, did not experience an excess severe toxicity or a poorer outcome compared with non-obese women treated similarly [87]. Later, a larger study showed that severe obesity was independently related with a lower likelihood of febrile neutropenia among those patients having full weight-based doses [82]. However, recent results from the GAIN (German Adjuvant Intergroup Node-positive) randomized phase III adjuvant trial showed an increased rate of severe toxicities (i.e., especially high-grade hematological events) in obese patients receiving full dose-dense chemotherapy regimen without influencing survival [88], further highlighting the necessity of a correct dose adjustment to avoid life-threatening complications in this group of patients. On the other hand, it is important to consider that most patients treated with chemotherapy have likely an increased chance of gaining weight [89,90].

The optimal use of endocrine therapy in the WHO-classified obese individuals is also an open and active area of research investigation, since several publications have described conflicting results about these therapy modalities as a function of BMI. Indeed, three randomized clinical trials have compared aromatase inhibitor versus tamoxifen treatment effectiveness in adjuvant breast cancer settings for all BMI categories: the ATAC (Arimidex, Tamoxifen Alone or in Combination) and the BIG (Breast International Group) 1–98 trials conducted in postmenopausal women, and the ABCSG (Austrian Breast and Colorectal Cancer Study Group) 12 trial in premenopausal ones. The ATAC study showed a lesser benefit of anastrozole versus tamoxifen in obese women compared to thinner patients [91], suggesting that estrogen suppression may be relatively incomplete in heavier individuals. Attenuated efficacy of the non-steroidal aromatase inhibitor anastrozole has also been reported in premenopausal obese women with early breast cancer treated with ovarian suppression by goserelin in the ABCSG 12 trial [92]. Specifically, overweight/obese women treated with anastrozole plus goserelin experienced a nearly 50% increase in the risk of disease recurrence and a three-fold higher risk of death compared with those patients that received tamoxifen plus goserelin. In contrast, interaction effects between BMI and treatment groups were not statistically significant at 8.7 years of median follow-up in the BIG 1–98 trial [93]. Moreover, the NSABP B14 (National Surgical Adjuvant Breast and Bowel Project) study, a National Cancer Institute-sponsored multicenter cooperative clinical trial, found that tamoxifen efficacy in women with lymph node-negative and ER-positive breast cancers did not vary across all BMI groups [94]; whereas an analysis evaluating raloxifene in the MORE (Multiple Outcome Raloxifene Evaluation) study showed a larger risk reduction with this treatment arm in women with a high BMI [95].

Although all of these studies add other pieces of evidence in an unfinished puzzle, more prospective trials are expected to further optimize breast cancer therapy in obese women, likely stratifying patients according to their different adipose depots.

### 2.5. Effects of Obesity on Quality of Life in Survivors

Obesity, at the time of breast cancer diagnosis and after receiving it, significantly impairs the quality of life and the functional health for survivors. Indeed, a greater percentage of overweight and obese patients reported sexual dysfunction, urinary incontinence, neuropathy, persistent physical fatigue, cardiotoxicity, and lymphedema compared with normal-weight women [96,97,98]. Conversely, an increased level of physical activity in a group of 692 breast cancer survivors with high BMI was associated with a better quality of life across various scales and dimensions [99]. More recently, results from the Women’s Health Initiative highlighted that postmenopausal women who decreased diet quality, as defined by the Healthy Eating Index (HEI)-2010 score, exhibited an increased risk of mortality [100]. In contrast, a cross-sectional analysis of pilot study data evidenced no significant association between (HEI)-2010 score and quality of life in survivors of early-stage breast cancer [101]. A number of ongoing trials, such as the German SUCCESS-C, the DIANA (Diet and Androgens)-5 and the BWEL (Breast Cancer Weight Loss) studies will provide conclusive evidence regarding the possibility of implementing weight loss interventions in breast cancer populations.

In the meanwhile, as part of standard care, oncologists should recommend for all breast cancer survivors to achieve and keep an ideal healthy body weight with an appropriate diet and a regular exercise.

## 3. Biological Mechanisms Linking Obesity to Breast Cancer

**Figure 1:**
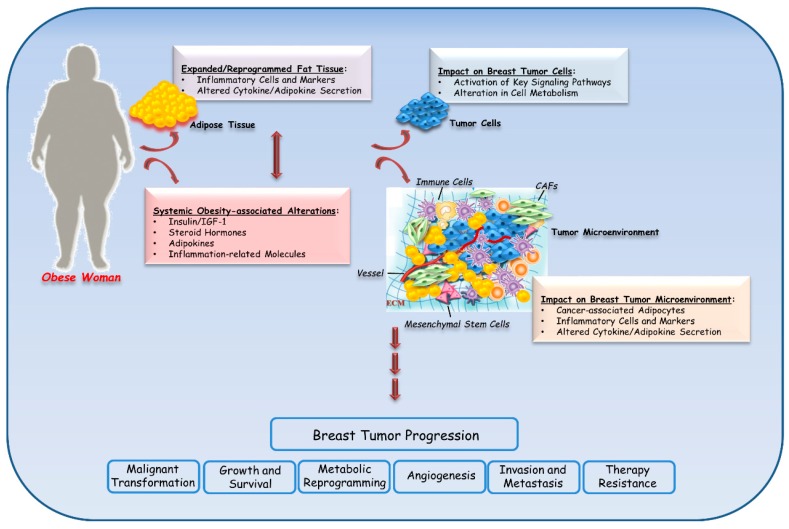
Mechanisms linking obesity with breast cancer. In obese women, excess adiposity is associated with key local and systemic changes, such as altered secretion of cytokines, adipokines, growth factors, and inflammatory molecules. The complex interplay among all of these alterations may contribute to breast cancer progression by both directly impacting the phenotype of cancer epithelial cells and indirectly affecting the behavior of the tumor microenvironment. IGF: insulin-like growth factor; CAFs: Cancer-associated fibroblasts; ECM: Extracellular Matrix.

Although the tight epidemiological connection between cancer development and obesity is well documented, the molecular mechanisms underlying the obesity-breast cancer link are still under investigation due to the complexity of obesity condition and the different oncogenic alterations that may sustain all breast cancer subtypes. Obesity is characterized by an expanded, metabolically active and reprogrammed fat tissue that induces local inflammation and altered levels of cytokines/adipokines. These local alterations contribute to and cooperate with systemic physiological changes regarding the levels of insulin, insulin-like growth factor (IGF) I, steroid hormones, adipokines, and inflammation-related molecules. Furthermore, hypertrophy and hyperplasia of white adipocytes reduces their vascularization, resulting in a decrease of the oxygen availability. This hypoxia state induces an increased oxidative stress, insulin resistance, ischemia, adipocyte necrosis and release of inflammatory as well as angiogenic proteins (reviewed in [51]). The local and systemic alterations induced by obesity may influence breast cancer through direct effects on neoplastic epithelial cells as well as indirect effects on tumor microenvironment. All these obesity-related factors can impact tumor initiation, metabolic reprogramming, angiogenesis, progression, and/or response to therapy (Figure 1). Interestingly, a balanced and healthy diet might contribute to down-regulating the expression of all these factors, providing a better outcome in obese patients with breast cancer [102].

### 3.1. Inflammation

It has been well recognized that chronic inflammation represents a pathophysiological condition that bridges obesity and cancer. Clinically, a positive association between circulating levels of C-reactive protein (CRP), a well-known marker of active inflammation, and postmenopausal breast cancer risk was found among women with excess adiposity [103]. In women diagnosed with primary breast cancer recruited to the HEAL (Health, Eating, Activity, and Lifestyle) study, serum levels of the inflammatory biomarkers amyloid A and CRP were associated with reduced disease-free survival [104]. An enrichment of several oncogenic inflammation-related pathways in obese patients compared to non-obese patients has also been identified in a transcriptomic analysis of human ERα-positive breast cancer samples [105].

Adipose tissue houses a unique immune cell repertoire and, during weight gain, an enhanced recruitment of adipose tissue macrophages (ATMs) along with a phenotypic switch towards an M1 proinflammatory state of ATMs may occur [106,107]. These events are concomitant with the production of inflammatory cytokines and mediators, such as tumor necrosis factor (TNF), interleukin (IL)-6 and -1β, and ROS generation, that contribute to local and systemic inflammation. Most of these factors were found at high levels in obese patients and were associated with poor outcome in breast cancer patients (reviewed in [108]). Moreover, in breast adipose tissue, infiltrating macrophages might surround and phagocytose damaged or necrotic adipocytes to form a syncytial arrangement, known as crown-like structures of the breast (CLSs). Macrophages constituting CLSs in adipose tissue have been associated with nuclear factor-kappa B (NFκB) activation and increased levels of several proinflammatory mediators, thus creating a positive feedback loop to further sustain chronic inflammation [109,110]. Importantly, CLSs are present in breast tissues of breast cancer patients, are enriched in overweight and obese patients, and negatively affect disease recurrence and survival [111,112,113].

More recently, a role for inflammasome as an important source of inflammation in the adipose tissue of obese subjects has also been described. Inflammasome, a macromolecular complex composed of Nod (Nucleotide-binding and oligomerization domain)-like receptors (NLRP1, NLRP3, and NLRC4), the adaptor apoptosis-associated speck-like protein containing a caspase-recruitment domain (ASC), and the enzyme caspase-1 (CASP-1), is a novel innate immune pathway required for triggering the maturation of proinflammatory cytokines, including IL-1β and IL-18 [114]. Adipocytes express multiple inflammasome related genes, including Nod-like receptor pathway genes [*NLRP3* and *PYCARD* (PYD-PYRIN-PAAD-DAPIN and CARD-caspase-recruitment Domain Containing)], *CASP1*, and other TLR (Toll-like receptor)-regulated genes [*IL1B*, *CCL2* (*C-C Motif Chemokine Ligand 2*), and *TNF*] and this adipocyte signature significantly increased in obese versus lean subjects [115]. Activated inflammasome and increased IL-1β production in tumor-associated macrophages generated an inflammatory microenvironment that promoted tumor growth and metastasis in animal and human breast cancer models [116]. Moreover, Kolb et al. proposed a novel molecular link between obesity and breast cancer involving NLRC4 inflammasome/IL-1β pathway activation in macrophages and the consequent increased angiogenesis through an up-regulation of vascular endothelial growth factor A (VEGFA) in adipocytes [117]. The complicated function of inflammasomes raises new challenges for the treatment of obese breast cancer patients.

### 3.2. Insulin-IGF-1 Axis

Insulin resistance, hyperinsulinemia, and/or hyperglycemia characterize patients with BMI ≥30 kg/m^2^ and are now considered as hallmarks of the obese state, leading to coin the term of “diabesity”. Insulin levels were significantly associated with increased breast cancer risk in a large prospective cohort of postmenopausal women [118]. Elevated nonfasting C-peptide levels, a surrogate marker for pancreatic insulin secretion, were associated with a higher breast cancer risk among women above 60 years of age within the EPIC (European Prospective Investigation into Cancer and Nutrition) study [119]. In contrast, a meta-analysis of observational studies did not find any association in studies adjusted for BMI and other factors [120]. A pooled individual data analysis of 17 prospective studies has also shown a positive association between high IGF-I levels and breast cancer risk, irrespective of menopausal status [121]. Besides breast cancer risk, insulin and IGF-1 levels have been positively connected to recurrence and mortality [122,123,124]. In addition, activated insulin and IGF-I receptors were detected in all breast cancer subtypes and associated with poor survival in patients [125].

The IGF-I signaling cascade has been shown to mediate breast cancer cell proliferation, migration, angiogenesis, survival, and resistance to therapy in different experimental “in vitro” and “in vivo” breast cancer models (reviewed in [126]). The significance of the IGF-IR pathway for breast cancer progression has led to the development of several inhibitors directed against this target and to the initiation of different clinical trials during the last decades [127,128].

### 3.3. Estrogens

In obese patients, predominantly but not exclusively associated to postmenopausal status, breast cancer risk is also related to elevated circulating sex steroid levels.

Few studies have evaluated the relationship of circulating concentrations of sex steroid hormones with breast cancer risk in premenopausal women; thus, data are inconsistent and further evaluations are still required. Indeed, a work published in 2013 in Lancet Oncology reported a strong association of circulating estradiol, calculated free estradiol, estrone, androstenedione, dehydroepiandrosterone, and testosterone with the risk of developing breast cancer in premenopausal women [129]. A case-control study nested within the EPIC cohort indicated an association only for increased serum testosterone levels [130]. On the contrary, epidemiological studies and meta-analyses have extensively reported that the estrogenic milieu in obesity is an important risk factor for postmenopausal women with ER-positive disease [45,131,132,133]. Among postmenopausal women, estrone, estradiol, and free estradiol levels were significantly associated with increasing BMI [134,135,136,137] and this relationship may be modified by weight loss and physical activity [137,138]. Importantly, beyond elevated circulating estrogen levels, the enhanced local production of this hormone, due to aromatase conversion from androgens, has been recognized as a crucial mechanism by which increased body weight may promote breast cancer development in postmenopausal women [139]. Indeed, an impressively higher mass of breast adipose tissue in obese individuals may increase estrogen biosynthesis within the breast because of both a higher number of aromatase-expressing fibroblasts and an increased aromatase expression per unit adipose tissue or cell [140]. Obesity may trigger different signaling pathways to induce aromatase gene expression/activity in adipose tissue of the breast and elsewhere, including breast tumor cells [141,142,143,144,145,146]. Then, the biologically active estradiol by binding to ERα facilitates “classical” and “nonclassical” genomic activities as well as rapid “non genomic” effects of the receptor to regulate downstream target protein expression involved in cell division, survival, angiogenesis, and invasion in breast cancer [147].

### 3.4. Adipokines

**Figure 2:**
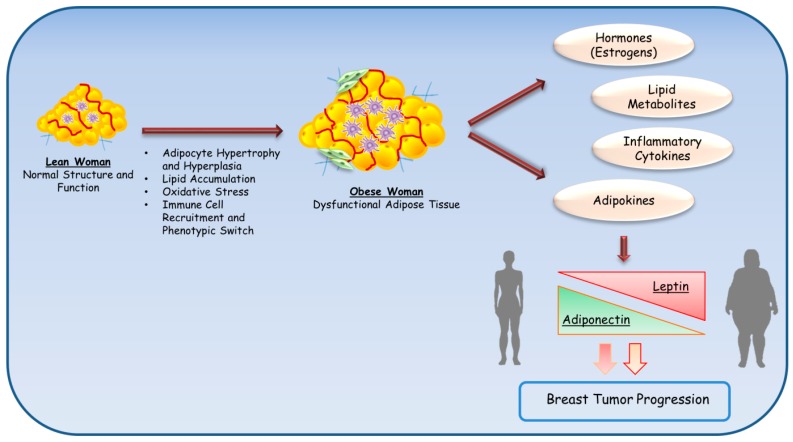
Adipose tissue alterations during weight gain. Obese adipose tissue expansion, characterized by adipocyte hypertrophy and hyperplasia, lipid accumulation, increased oxidative stress, and immune cell infiltration (i.e., macrophages), leads to the development of a dysfunctional adipose tissue which produces a number of bioactive molecules, such as sex hormones, lipid metabolites, and proinflammatory cytokines. In obesity, fat accumulation also causes a dysregulation of adipokine production, resulting in increased leptin and decreased adiponectin levels that strongly contribute to the onset of obesity-associated breast cancers.

The pathological expansion of white adipose tissue in obesity leads to the development of a dysfunctional adipose tissue which is associated with an abundant production of several biologically active factors, including hormones, lipid metabolites and inflammatory cytokines, along with an altered adipokine profiles [148,149]. Adipokines, a large group of heterogeneous peptides mainly produced by adipose tissue, are emerging as key molecules linking obesity to cancer. Currently more than 100 different adipokines have been identified, and among these, leptin and adiponectin have come to be recognized for their influence on breast cancer risk and tumor biology (Figure 2).

#### 3.4.1. Adiponectin

Adiponectin, whose circulating levels are inversely correlated with adiposity, is a multifunctional protein with pleiotropic effects on several tissues and organs and has been proposed to exert protective roles against the development of obesity-related disorders, such as metabolic syndrome, diabetes, cardiovascular diseases and malignancies [150]. Adiponectin, the most abundant adipose-tissue protein, is a 244-amino acid polypeptide mainly secreted by white adipose tissue, but it is also produced at lower concentrations by brown fat, skeletal muscle, cardiomyocytes, liver, bone marrow and cerebrospinal fluid [150]. The circulating and intracellular forms of adiponectin (low, medium, high molecular weight and globular) and the tissue-specific expression of adiponectin receptors (AdipoR1, AdipoR2, and T-cadherin) influence different cellular processes involved in tumorogenesis. AMPK/LKB1 signaling, the main pathway of adiponectin action, is involved in the regulation of apoptosis, proliferation, angiogenesis and energy metabolism. In particular, once activated, the receptor promotes the translocation of LKB1/STE20-related adaptor protein (STRAD)/scaffolding mouse 25 protein (MO25) from the nucleus to the cytoplasm and induces LKB1 phosphorylation with concomitant activation of AMPK and consequent inhibition of MAPK, PI3K/Akt, WNT-β-catenin, NFκB, and JAK2/STAT3 signaling [151,152].

It has been reported that adiponectin negatively influences breast carcinogenesis, although conflicting results have been published. Many clinical investigations suggested that low adiponectin concentrations are associated with an increased risk of breast cancer (OR (Odds Ratio) = 3.63 [153] and OR = 0.84 [154]) and such an interaction has also been reported with menopausal status (RR = 0.73 among postmenopausal women and 1.30 for premenopausal women [155] and SRR (Standardized Relative Risk) = 0.80 and =0.72 in post and premenopausal patients, respectively [156]). Serum adiponectin concentrations showed an inverse association with breast cancer recurrence in ER/PR-negative patients [157]. Adiponectin expression was significantly correlated with smaller tumor size and lower T-stage in invasive breast cancer [158]. Several “in vitro” and “in vivo” studies have shown that adiponectin induces cell growth arrest and apoptosis, suppresses cell proliferation, invasion and migration in ER-negative breast cancer cells [159,160,161,162,163]; whereas controversial observations have been reported on its effects on ER-positive breast cancer cells (reviewed in [164]). Recent data have suggested that low levels of adiponectin, similar to those found in obese women, may differently impact breast cancer progression in relation to ERα expression. Indeed, it has been discovered, in ER-positive breast cancer cells, that low adiponectin concentrations promote the interaction of APPL1 with AdipoR1, ERα, IGF-IR, and c-Src that is responsible for MAPK phosphorylation. This induced activation of ERα at genomic levels, thus contributing to breast cancer growth [165]. In addition, adiponectin, by differently modulating cyclin D1 expression (i.e., down-regulation in ERα-negative cells and up-regulation in ERα-positive cells) caused divergent effects on cell proliferation [166]. More recently, it has also been demonstrated that ERα/LKB1 interaction may negatively interfere with the ability of LKB1 to phosphorylate AMPK, thus inhibiting its downstream mTOR signaling. In contrast, in ER-negative breast cancer cells the interaction of LKB1 with AMPK resulted in mTOR inhibition, thus negatively regulating breast cancer cell proliferation [167]. Therefore, targeting adiponectin signals might be of value in patient management, but in relation to specific breast cancer subtypes.

#### 3.4.2. Leptin

Leptin, whose synthesis and plasma levels increase in proportion to fat mass, is a 16 kDa multifunctional polypeptidic molecule encoded by the obese (*Ob*) gene. Besides its neuroendocrine function, leptin can affect a wide range of biological activities, including mammary tumorogenesis. Indeed, it has been extensively demonstrated that this adipokine, mainly produced by distant and local adipocytes but also by epithelial tumor cells itself and by other cells within the tumor-stroma (i.e., cancer associated fibroblasts), is able to affect different aspects of breast cancer biology in an endocrine, paracrine and autocrine manner [168,169,170,171,172,173]. The molecular actions of leptin are mediated through the transmembrane leptin receptor (ObR) encoded by *db* gene, a member of the class I cytokine receptor family ubiquitously expressed in several tissues. Leptin binding to the long ObR isoform induces activation of several intracellular downstream signaling pathways, such as JAK2/STAT3, MAPK and PI3K/Akt pathways involved in the control of cell proliferation, differentiation, survival, migration, and invasion. Indeed, several data from clinical and experimental studies strongly support the involvement of leptin in mammary tumor development and progression.

##### Clinical Studies

Although different epidemiological studies have reported contradictory results regarding the association between leptin concentrations and breast cancer, a meta-analysis of 23 studies has shown that leptin levels are positively associated with breast cancer risk [174]. Specifically, Niu et al. reliably stated that circulating leptin levels are different among several population groups from low to high as follows: healthy people < breast benign disease patients < breast cancer patients < lymph node metastasis-positive patients, suggesting that the assessment of leptin levels should be considered as a suitable diagnostic tool in this neoplasia. Recently, other two studies further highlighted the potential role of leptin as a biomarker for breast cancer risk, especially in overweight/obese subjects and postmenopausal women [175,176]. Kaplan–Meier survival analysis also indicated that high ObR expression correlated with a reduced rate of overall survival in breast carcinoma patients, with a more relevant discrimination for basal-like breast cancer subtypes [173]. Moreover, leptin and its receptor were found to be overexpressed in breast cancer, especially in higher grade tumors and were associated with distant metastasis and poor prognosis [177,178,179,180]. Remarkably, weight loss interventions through diet and exercise in overweight/obese breast cancer survivors were associated with a decrease in specific biologic factors related to breast cancer recurrence and mortality, such as estrogen, insulin, and leptin levels [5,67].

##### In Vivo Studies

Many “in vivo” studies have attempted to define the role of obesity and leptin in impacting breast cancer. Zucker rats, bearing a leptin receptor missense mutation with consequent absence of leptin response, are recognized as a model to reproduce obese metabolic syndrome. Following administration of the chemical carcinogen methylnitrosourea, the development of a smaller percentage of breast carcinomas was observed in obese Zucker rats compared with lean controls [181]. In addition, it has been also reported that other two well-recognized standard genetic models of obesity, including mouse mammary tumor virus (MMTV)-TGF-α/Lep(ob)/(ob) (leptin-deficient) and MMTV-TGF-α/Lepr(db)/(db) (leptin receptor-deficient) mice, did not develop mammary tumors compared to wild-type mice [182,183]. However, these mouse models exhibited defective mammary gland development, representing a bias for studying the specific involvement of leptin in obese-induced cancers. In this respect, Park et al. showed that reconstitution of leptin-receptor signaling in the brain of db/db mice restored the development of the mammary gland [184]. These mice, with an intact central leptin signaling and a deficient peripheral leptin receptor, exhibited a decreased mammary tumor growth and progression. The importance of leptin signaling in breast carcinogenesis was then highlighted in another work, showing that this adipokine was able to sustain tumor progression in MMTV-Wnt-1 mice, while mammary tumor growth was inhibited in leptin-deficient mice (Lepob/ob) [185]. Recently, an increased tumor incidence and aggressiveness along with elevated leptin/leptin receptor expression and signaling activation were found in mammary tissues of rat model of breast cancer driven by western diet-induced obesity [186]. In addition, knockdown of leptin in adipose stromal/stem cells isolated from obese patients resulted in reduced tumor growth and numbers of lung and liver metastasis in SCID (severe combined immunodeficiency)/beige mice [187].

##### In Vitro Studies

**Figure 3:**
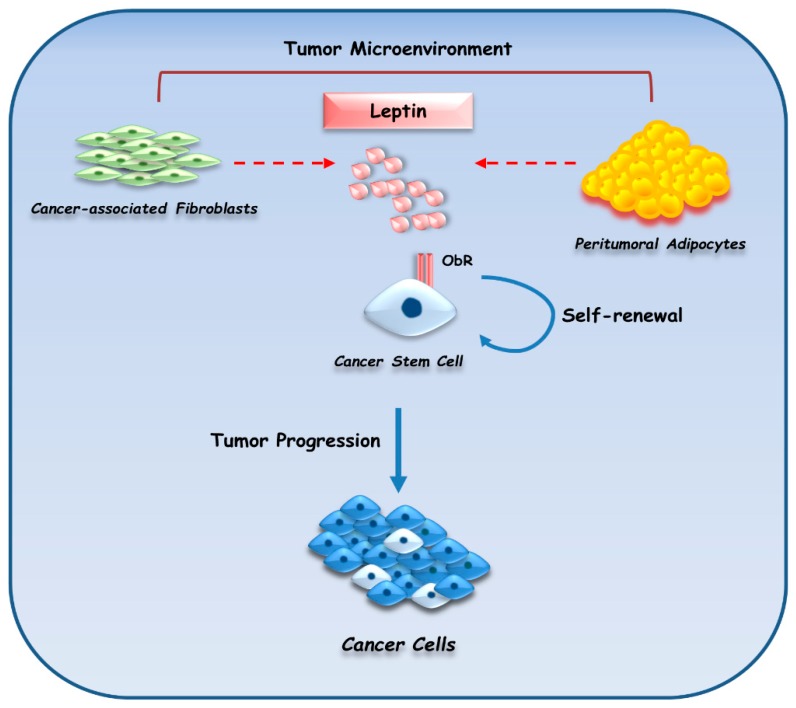
Role of leptin in mediating the interaction between stromal cells and breast cancer stem cells. Leptin, an important paracrine molecule secreted by adipocytes and cancer-associated fibroblasts within the tumor microenvironment, sustains breast cancer stem cell activity, indicating an additional mechanism by which this adipokine promotes breast malignancy.

In line with the “in vivo” findings, a growing body of experimental “in vitro” evidence clearly demonstrated the multifaceted role of leptin in supporting the oncogenic phenotype of breast cancer. Indeed, leptin has been shown to have several pro-tumorigenic effects, including increased cell proliferation, transformation, antiapoptotic effects, self-renewal and reduced efficacy of breast cancer treatments (reviewed in [168,169,170]). Besides, this adipokine shaped the tumor microenvironment by inducing the migration of endothelial cells, angiogenesis, and the recruitment of immune cells such as macrophages [168]. In addition to its direct action through its own receptor, leptin can crosstalk with other different signaling molecules, including ERα, growth factors, Notch, and inflammatory cytokines to further affect breast cancer risk, progression, recurrence and mortality. It has been reported that estradiol administration increases leptin and ObR expression in ER-positive MCF-7 breast cancer cell lines [178]. Interestingly, we identified leptin as a strong amplifier of the estrogen signaling and this occurred through a double mechanism: an up-regulation induced by leptin on aromatase gene expression together with its capability to directly transactivate ERα [143,188]. The biological significance of the synergy between leptin and ERα was highlighted by “in vivo” studies using xenograft models. Indeed, leptin exposure increased tumor volume and doubled tumor size after 13 weeks compared to estradiol treatment [189]. We have also demonstrated that a lysine to arginine mutation at residue 303 (K303R) within the hinge domain of ERα may potentiate ERα’s role as an effector of leptin signaling, which results in an increased cell proliferation, migration, and invasion, thus contributing to the more aggressive phenotype of K303R-associated breast cancers [172]. Leptin also interfered with the estrogen antagonist ICI 182,780 action by reducing its effects in MCF-7 breast cancer cells [190]. Different studies have reported an interplay between leptin and different members of growth factor family, resulting in an enhanced growth and metastatic properties of breast cancer cells [191,192,193]. In this context, we have shown that leptin, by inducing heat-shock protein 90 (Hsp90) expression, enhances the membrane tyrosine kinase receptor HER2 protein levels and this results in a reduced sensitivity of breast cancer cells to antiestrogen tamoxifen treatment [194]. Moreover, a complex signaling network between Notch, IL-1, and leptin (NILCO), described by Gonzalez-Perez’s group, has been shown to drive breast tumorigenesis. Particularly, this crosstalk represents the integration of developmental, pro-inflammatory and pro-angiogenic signals critical for leptin-induced cell proliferation, migration, angiogenesis and self-renewal of breast cancer stem cells (CSCs) [195,196]. CSCs play crucial roles in tumor initiation, metastasis and therapeutic resistance and the involvement of leptin in CSC survival has also been proposed in other studies. Indeed, selective expression of leptin receptor has been considered as a feature of CSCs, which exhibited an increased response to leptin, including phosphorylation and activation of STAT3 and induction of stem cell markers, such as OCT4 and SOX2 [197]. Moreover, several studies have indicated that the adipokine leptin regulates many signaling pathways (i.e., Notch, Wnt, mTOR, STAT3, HER2/Erb, and IGF pathways) and transcription factors (i.e., NFκB and hypoxia-inducible factor) which are critically implicated in breast CSC activity [195,198,199,200,201]. Chang et al. demonstrated that leptin, through STAT3 signaling, recruits G9a histone methyltransferase to the STAT3 response element located within the 2-kb region upstream of the transcription starting site of miR-200c. This caused a repression of miR-200c by epigenetic silencing and consequently promoted the formation of breast CSCs [186]. Inhibiting the STAT3/G9a pathway restored expression of miR-200c, which, in turn, reversed the CSC phenotype. Besides, in a rat model of breast cancer driven by diet-induced obesity, treatment with a specific STAT-3 inhibitor (S3I-201) significantly reduced the tumor sphere-forming capacity and decreased tumor growth [186]. Moreover, Wang et al. have shown that leptin-JAK/STAT3 signaling activates fatty acid b-oxidation (FAO), through enhanced transcription of carnitine palmitoyltransferase 1B (CPT1B), thus promoting breast cancer stemness and chemoresistance. Accordingly, targeting FAO and/or depleting leptin inhibited breast CSCs reversed chemoresistance and reduced breast tumor growth [202]. In line with these observations, our recent findings demonstrated a direct involvement of leptin and its receptor in mediating the interaction between stromal cells (cancer-associated fibroblasts and breast adipocytes) and breast CSCs, providing novel insights into understanding how breast CSCs are influenced by the tumor microenvironment (Figure 3) [173]. Interestingly, blocking leptin signaling by using a full leptin receptor antagonist, the peptide LDFI [203], completely reversed the breast CSC phenotype [173], further highlighting the potential advantage of targeting leptin signaling to block breast cancer malignancy.

## 4. Conclusions and Perspective

The epidemic of obesity is now recognized as one of the most significant public health concerns facing the world nowadays. Pathological expansion of white adipose tissue and high levels of certain cytokines and leptin, as a consequence of the obesity condition, have been implicated in several hallmarks of breast cancer, such as inflammation, sustained proliferative signaling, epithelial-to-mesenchymal transition, angiogenesis, and cellular energetics. However, our understanding of the weight of obesity on breast cancer pathogenesis is only beginning to influence routine clinical practice. Although ongoing studies are focusing on interventions for both primary and secondary prevention of obesity-related breast cancers, key biomarkers of risk are still lacking. Furthermore, whether and how the myriad of local and systemic effects of obesity may impact patient outcome and response to conventional/targeted treatments is an issue that requires additional investigation in experimental models and in humans. As with the development of more personalized oncology approaches, the evaluation of novel therapeutic strategies directed against the mechanisms described above (e.g., leptin-targeting agents) is also urgently needed in at-risk obese populations.

## Figures and Tables

**Table 1:** List of the studies on obesity and breast cancer with ≥350 participants at www.clinicaltrials.gov.

Clinicaltrials.gov	Interventions	Status	Eligible Criteria, Primary Outcome and Purpose	Results
**NCT01758146**	Drug: Tamoxifen Drug: Letrozole	Phase 3	**Eligibility criteria:** Postmenopausal patients (45–80 years) with hormone receptor positive breast cancer (tumor stage IB, IC, or II irrespective of nodal stage (<10 positive nodes)). **Primary outcome:** Disease free survival (time frame: from date of random assignment to the first event over 5 years). Event in the form of locoregional recurrence, distant metastasis, cancer in the contralateral breast, second primary cancer, or death from any cause. **Purpose:** To see the impact of obesity on the efficacy of adjuvant endocrine therapy with aromatase inhibitors in postmenopausal patients with early breast cancer in terms of: (1) Locoregional recurrence; (2) Distant metastases; (3) Disease-free survival; (4) Overall survival.	Not applicable
**NCT00470119**	Behavioral: Behavioral dietary intervention Behavioral: Exercise intervention	Not applicable	**Eligibility criteria:** Postmenopausal patients (50–75 years) with no history of invasive or “in situ” breast cancer and at increased risk for developing breast cancer due to several lifestyle risk factors (e.g., lack of physical activity, excess weight, obesity, etc.). **Primary outcome:** Serum estrone concentrations as measured by radioimmunoassay (time frame: at baseline and 12 months) **Purpose:** To compare the effects of a 1-year exercise intervention, reduced-calorie diet intervention, or a combined exercise and reduced-calorie diet intervention vs. no intervention on serum estrone concentrations in overweight or obese postmenopausal women.	[17,18,19,20,21,22,23]
**NCT01172886**	Not applicable	Unknown	**Eligibility criteria:** Healthy women and women operated for breast cancer (35–80 years). **Primary outcome:** Biospecimen retention: Samples without DNA. Blood samples. **Purpose:** To evaluate if the adjustments in lifestyle with physical activity intensification and healthy diet may represent modifiable factors on which sporadic breast cancer primary prevention may work.	Not applicable
**NCT02750826**	Other: Health education program Other: Weight loss intervention	Phase 3	**Eligibility criteria:** Subjects (≥18 years) with histologically confirmed invasive breast cancer and with registration within 12 months after the first histologic diagnosis of the disease.**Primary outcome:** Invasive disease-free survival (time frame: up to 10 years).**Purpose:** To compare the effect of a supervised weight loss intervention plus health education materials versus health education materials alone on invasive disease-free survival (IDFS) in overweight (body mass index, BMI 27–29.9 kg/m^2^) and obese (BMI ≥30 kg/m^2^) women diagnosed with HER-2 (Human epidermal growth factor receptor 2) negative, stage II, and III breast cancer.	Not applicable
**NCT01112839**	Behavioral: Less intensive Behavioral: Intensive group	Completed	**Eligibility criteria:** Patients (≥21 years) with breast cancer (stages I (≥1 cm), stage II, or stage IIIA, B, C excluding distant metastasis) diagnosed between 6 months and 5 years earlier; after initial therapies were completed. BMI between 25 and 45 kg/m^2^. **Primary outcome:** Weight loss (time frame: 2 years). **Purpose:** To explore whether two different programs that are focused on weight management, through increased exercise and a healthy diet, are feasible, and have an impact on body weight, quality of life and fatigue.	[24]
**NCT02618538**	Not applicable	Enrolling by invitation	**Eligibility criteria:** All women (46–67 years) undergoing a screening mammography in the participating centers (Turin, Vercelli, and Biella). **Primary outcome:** Positive predictive value (PPV) of breast cancer risk factors (time frame: 2 years). **Purpose:** To estimate in a large cohort of women attending breast cancer screening, the predictive positive values of different breast cancer risk factors (alone or in combination) in order to identify appropriate risk-based stratifications for personalized screening.	[25]
**NCT00393172**	Behavioral: exercise	Phase 2 Phase 3	**Eligibility criteria:** Premenopausal healthy women (18–30 years). **Primary outcome:** Changes in urine levels of F2-isoprostanes (time frame: before and after study). **Purpose:** To compare the changes in urine levels of a stable marker of oxidative stress (F_2-isoprostanes) in healthy young women who undergo exercise training during 4 menstrual cycles vs. no exercise.	[26,27,28,29]
**NCT00509626**	Behavioral: exercise intervention Other: questionnaire administration Procedure: CAM (Complementary and Alternative Medicine) exercise therapy Procedure: management of therapy complications Procedure: psychosocial assessment and care Procedure: quality-of-life assessment	Phase 1 Phase 2	**Eligibility criteria:** Premenopausal and postmenopausal patients (≥ 18 years) with diagnosis of primary breast cancer and BMI ≥20 kg/m^2^ and <39 kg/m^2^. **Primary outcome:** Accrual (phase I); Retention (phase I); Weight change after 6 months (phase II). **Purpose:** To determine whether: (1) conducting a clinical trial that uses a 6-month physical activity intervention initiated within 45 days after surgery for early-stage breast cancer and prior to initiation of adjuvant chemotherapy, hormonal therapy, and/or radiotherapy is feasible (phase I); (2) the participation in a 6-month physical activity intervention initiated within 45 days after surgery for early-stage breast cancer decreases weight gain in patients treated with adjuvant chemotherapy and differentially affects weight change in women who are premenopausal compared to those who are postmenopausal at diagnosis (phase II).	Not applicable
**NCT03529383**	Device: Connected device Behavioral: Therapeutic education	Not applicable	**Eligibility criteria:** Patients (18–75 years) operated for a first primary non-metastatic invasive breast carcinoma, with the prescription of an adjuvant treatment (chemotherapy, hormonotherapy, radiotherapy). **Primary outcome:** Number of patients achieving the internationally recommended level of physical activity of at least 150 min per week of moderate-to-vigorous physical activity [intensity ≥3 METs (Metabolic equivalent of task)] (time frame: 6 months). Assessment by the RPAQ self-administered questionnaire. **Purpose:** To identify best modalities to implement exercise during and after breast cancer treatment. In a public health perspective, the challenge is to reduce geographical, social, and organizational inequalities among patients to practice regular physical activity and to promote the systematic integration of physical activity in routine cancer care.	[10,13,30,31,32,33,34,35,36,37]
**NCT01707121**	Not applicable	Completed	**Eligibility criteria:** Patients (≥18 years) scheduled for cholecystectomy, breast cancer surgery, colorectal cancer surgery. **Primary outcome:** Length of sick-leave/time to work (time frame: 6 weeks) for breast cancer and gallbladder surgery. Length of hospital stay (time frame: 6 weeks] for colorectal surgery. **Purpose:** To investigate whether a higher physical activity prior to a surgical procedure reduces hospital stay, sick leave, and the complication rate.	[38]
**NCT02273206**	Behavioral: Preventive care management for depression Behavioral: Preventive care management for cancer screening	Not applicable	**Eligibility criteria:** Patients (50–64 years) from the Bronx with no cancer diagnosis within the past six months and overdue for breast, cervical, or colorectal cancer screening. **Primary outcome:** Changes from baseline in number of participants with colorectal, breast, and/or cervical cancer screening (time frame: 12 months).Chart Review: Review of electronic medical records at each of the three community health centers.Self-Report: We will ask participants about their participation (yes/no) in specific screening methods: Pap testing (past 3 years), mammography (past 2 years), and colorectal screening (FOBT (fecal occult blood test)/FIT (fecal immunochemical test), past year; flexible sigmoidoscopy, the past 5 years; and colonoscopy, past 10 years). Change from baseline in Patient Health Questionnaire (PHQ)-9 at 6 months (time frame: 6 months). The PHQ-9 is the 9-item depression module from the full PHQ, and is a well-validated Diagnostic and Statistical Manual of Mental Disorders 4th Edition (DSM-IV) criterion-based measure for screening and diagnosing depressive episode, assessing severity, and monitoring treatment response, either in person or over the phone.Change from baseline in Patient Health Questionnaire (PHQ)-9 at 12 months (time frame: 12 months). The PHQ-9 is the 9-item depression module from the full PHQ, and is a well-validated Diagnostic and Statistical Manual of Mental Disorders 4th Edition (DSM-IV) criterion-based measure for screening and diagnosing depressive episodes, assessing severity, and monitoring treatment response, either in person or over the phone.Change from baseline in The Hopkins Symptom Checklist (SCL-20) at 6 months (time frame: 6 months). The SCL-20 consists of 20 depression items on a 4-point scale from the SCL-90, and has been shown to be a valid and reliable measure of depression in diverse outpatient and community populations.Change from baseline in The Hopkins Symptom Checklist (SCL-20) at 12 months (time frame: 12 months). The SCL-20 consists of the 20 depression items on a 4-point scale from the SCL-90, and has been shown to be a valid and reliable measure of depression in diverse outpatient and community populations. **Purpose:** To increase the investigators understanding of how to enhance primary care systems’ ability to improve a range of outcomes related to cancer screening and depression among low-income minority women, and how to best support this population in making cancer-screening decisions.	Not applicable
**NCT01515124**	Behavioral: Exercise intervention Behavioral: Weight loss intervention	Not applicable	**Eligibility criteria:** Overweight or obese child, adult, or older adult female breast cancer survivor, at least 2 months after treatment (e.g., surgery, chemotherapy, or radiotherapy), currently free of cancer. **Primary outcome:** Clinical lymphedema exacerbation rate (time frame: Baseline, 6, and 12 months). Lymphedema outcomes include: incident events requiring medical care for lymphedema (e.g., flare-ups or cellulitic infections), arm swelling in the affected limb (interlimb volume differences), and pain and lymphedema symptoms (number and severity). **Purpose:** To test the effects of these interventions on lymphedema outcomes, breast cancer recurrence, and quality of life.	[39]
